# Nanomedicine-based cancer immunotherapy: translational barriers, mechanistic strategies, and future perspectives

**DOI:** 10.1080/10717544.2026.2679846

**Published:** 2026-05-27

**Authors:** Xiaoman Suo, Yingnan Liu, Guofang Zhang, Yang Li

**Affiliations:** a Laboratory of Inflammation and Vaccines, Shenzhen Institutes of Advanced Technology, Chinese Academy of Sciences, Shenzhen, Guangdong, People's Republic of China; b Laboratory of Immunology and Nanomedicine & China-ltaly Joint Laboratory of Pharmacobiotechnology for Medical Immunomodulation, Shenzhen Institutes of Advanced Technology, Shenzhen, Guangdong, People's Republic of China; c Division of Allergy & Immunology, Department of Biosciences & Medical Biology, Paris Lodron University of Salzburg, Salzburg, Austria; d State Key Laboratory of Biomedical Imaging Science and System, Shenzhen, Guangdong, People's Republic of China

**Keywords:** Nanomedicines, cancer immunotherapy, protein corona, nano-molecular specific interaction, artificial intelligence

## Abstract

Nanomedicine-based cancer immunotherapy integrates nanotechnology with immune modulation, representing a promising strategy to improve both the efficacy and safety of cancer treatment. Despite substantial preclinical potential, clinical translation is hindered by interconnected challenges in pharmacology, pharmacodynamics, and long-term safety. This mechanism-oriented prospective analyzes translational bottlenecks, pharmacological uncertainty from biomolecular corona, suboptimal pharmacodynamics due to tumor barriers, and metabolism/excretion affecting biosafety. Using a concept-driven framework, we link nano-bio interactions to clinical outcomes within a ‘barrier-strategy’ paradigm. Corresponding strategies such as mechanism-driven design, AI-assisted optimization, and advanced delivery systems are discussed, with emphasis on safety-by-design principles. Collectively, this perspective provides a forward-looking roadmap for future research, underscoring the importance of integrated technologies, advanced translational models, and scalable manufacturing to fully realize the clinical potential of nanoimmunotherapy.

## 1. Introduction

Cancer is a leading cause of illness and death globally. Traditional treatments like surgery, radiotherapy, and chemotherapy focus on directly killing tumour cells but often fall short due to incomplete tumour removal, systemic toxicity, drug resistance, and lack of specificity (Lisa et al. 2025). In contrast, cancer immunotherapy has revolutionised treatment by leveraging the immune system to identify and destroy cancer cells, providing systemic tumour control and long-lasting immune activation (Spotlight on cancer immunotherapies 2025). Notable advances include immune checkpoint blockade (ICB) targeting PD-1/PD-L1 (Programmed Cell Death Protein-1/Programmed Death-Ligand 1) and CTLA-4 (Cytotoxic T-Lymphocyte–Associated Protein 4), adoptive cell therapies like CAR-T (Chimeric Antigen Receptor T) cells, and cancer vaccines, showcasing the promise of immunotherapy (Sun et al. 2023). Despite these successes, current immunotherapies face challenges such as inefficient delivery to tumour sites, limited effectiveness against solid tumours, and systemic toxicity, including immune-related adverse events like colitis, dermatitis, hepatitis, endocrine disorders, and cytokine release syndrome (Xue et al. 2024).

Nanomedicine-based cancer immunotherapy, or nanoimmunotherapy, is a promising approach that combines advancements in nanotechnology, materials science, immunology, and oncology to address existing challenges (Grippin et al. 2025). By designing nanoscale systems (1-1000 nanometres) with adjustable physicochemical properties, nanoimmunotherapy can improve delivery precision, optimise pharmacokinetics and pharmacodynamics, and minimise off-target toxicity (Gomerdinger et al. 2025). These nanomedicines can function as carriers for immunomodulators or act as immunomodulators themselves, achieving passive accumulation, active targeting, and microenvironment-responsive release (Nano-enabled immunomodulation 2021; Gomerdinger et al. 2025). Despite significant progress in preclinical studies, clinical translation is limited. Few drugs have received regulatory approval, and many fail in late-stage clinical trials. This ‘translational gap’ is primarily due to incomplete understanding of nano-bio interactions, inefficient penetration of biological barriers, and unresolved long-term safety concerns (Q&A Translational Cancer Nanomedicine 2025). These issues underscore the urgent need to transition from empirical to mechanism-driven design strategies.

**Figure 1.**
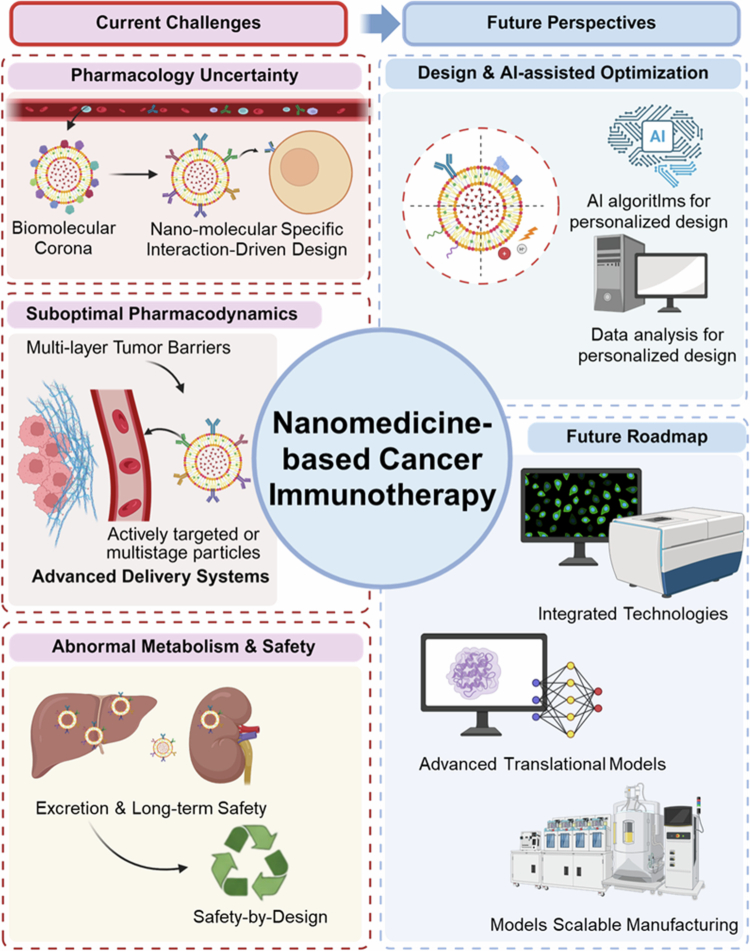
Schematic illustration of the current challenges and future perspectives of nanomedicine-based immuno-oncotherapy**.** The schematic is organised into two major sections. The left panel summarises three key challenges, including pharmacological uncertainty, suboptimal pharmacodynamics, and abnormal metabolism with long-term safety concerns. The right panel highlights two forward-looking aspects: design and AI-assisted optimisation, and a future roadmap toward clinical translation, encompassing integrated technologies, advanced translational models, and scalable manufacturing.

This perspective highlights the core value of nanoimmunotherapy, identifies major translational challenges, and outlines strategies to bridge the gap between lab innovations and clinical practice. We aim to create a coherent framework for designing next-generation nanomedicines and advancing their translation into viable cancer immunotherapies. To this end, the perspective is structured into three modules: translational barriers (pharmacological uncertainty, suboptimal pharmacodynamics, safety), mechanism-guided engineering strategies, and clinical progress with future directions. While these barriers underscore the complexity of nanoimmunotherapy, they also offer a foundation for rational design. Unlike material- or application-focused reviews, we adopt a mechanism-centred, translation-oriented perspective that links nano-bio interactions (corona formation, TME barriers) to pharmacokinetics/pharmacodynamics and safety within a ‘barrier-strategy’ framework. Encouraging preclinical advances call for rigorous clinical validation, with future efforts focused on mechanism-informed design, AI-driven optimisation, and clinically relevant models. Figure 1 summarises current challenges and future perspectives.

## 2. Core values and translational bottlenecks of nanoimmunotherapy

### 2.1. Core value proposition

Nano-immunotherapy is an interdisciplinary field focused on developing nanoscale treatment systems. Its primary goal is to address the limitations of traditional immunotherapy, such as poor penetration into solid tumours, inadequate intratumoral drug concentration, significant off-target toxicity, and low efficacy in activating anti-tumour immunity (Pan et al. 2024). Current nanotechnology-based immunotherapy platforms focus on several key strategies. These include the development of nanocarriers for the delivery of immunotherapeutic payloads, enabling the encapsulation and controlled release of bioactive agents such as antibodies, cytokines, and nucleic acids. In addition, nanomaterials are engineered to possess both adjuvant properties and intrinsic immunomodulatory activities, allowing the construction of nanocarrier-based combination therapy systems. Finally, nanotechnological approaches are employed to enhance adoptive cell therapy by improving cell viability, functionality, and therapeutic efficacy (Nano-enabled immunomodulation 2021; Song et al. 2021; Xue et al. 2024; Linderman et al. 2025).

Nanoimmunotherapy offers a distinct advantage by enabling precise modulation of the physicochemical properties of nanomaterials, thereby facilitating efficient and controlled immunomodulation (Lee et al. 2023). By adjusting particle size, shape, charge, and surface ligands, nanocarriers can exploit the enhanced permeability and retention (EPR) effect for passive targeting and use ligand-receptor interactions for active targeting of tumour or specific immune cells. This dual targeting increases drug accumulation at the lesion site while minimising systemic toxicity (Chen et al. 2025). Additionally, nanocarriers can respond to tumour microenvironment features, such as acidic pH, abnormal enzyme activity, and hypoxia, or external stimuli to achieve spatiotemporally controlled therapeutic release, enhancing immune activation precision (Mi 2020). Their ability to co-deliver multiple payloads protects fragile immunotherapeutic molecules, improves in vivo stability and bioavailability, and synergistically activates innate and adaptive immune responses (Peng et al. 2022). This reshapes the tumour immune microenvironment, converting ‘cold tumours’ into ‘hot tumours.’ Taken together, these features highlight the capacity of nanoimmunotherapy to achieve precise spatiotemporal regulation of antitumor immune responses through the integration of targeted delivery, controlled release, and multifunctional synergy (Gao et al. 2021; Song et al. 2021).

### 2.2. Multidimensional barriers to the clinical translation of nanoimmunotherapy

**Figure 2.**
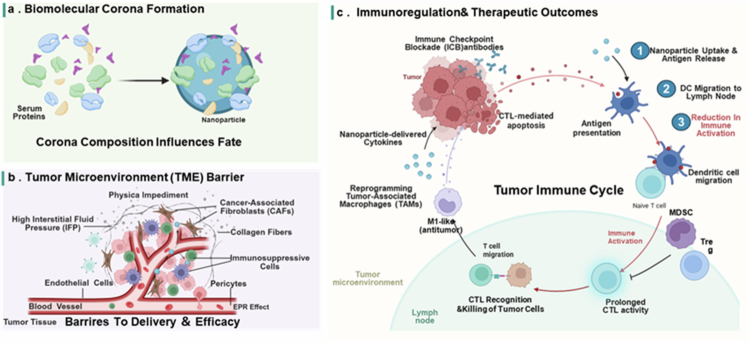
Biological processes governing nanoparticle-based cancer immunotherapy. (a) Biomolecular corona formation on nanoparticles. The adsorption of serum proteins forms a biomolecular corona that determines the biological fate of nanoparticles. (b) Tumour microenvironment (TME) barriers to nanoparticle delivery. High interstitial fluid pressure, dense stroma (collagen, CAFs), and immunosuppressive cells limit nanoparticle penetration and efficacy, despite the EPR effect. (c) Nanoparticle-mediated immunomodulation of the tumour-immune cycle. Nanoparticles enhance cancer immunotherapy by promoting antigen release, DC activation, and CTL-mediated tumour killing, while also delivering ICB agents, reprogramming TAMs, and suppressing immunosuppressive cells.

Both pharmacodynamic (PD) and pharmacokinetic (PK) processes are critical for understanding the multidimensional barriers to clinical translation in nanoimmunotherapy. PD covers downstream immune activation and outcomes. PK covers the enrichment of the tumour microenvironment. Nano-protein interactions affect both: PK uncertainty arises from corona formation, biodistribution, and clearance; PD unpredictability from corona-modulated immune recognition and uptake, beyond TME heterogeneity (Figure 2). These PK/PD barriers and safety form the multidimensional landscape hindering clinical translation.

#### 2.2.1. Uncertainties in pharmacological mechanisms

Pharmacological uncertainty primarily involves the unpredictable interactions between nanomaterials and biological systems at the molecular and cellular levels. Unlike small-molecule drugs with specific targets, nanomedicines depend on complex, multi-step processes, complicating the clear understanding of their mechanisms (Wu et al. 2025). A critical factor is the formation of protein coronas: once nanoparticles enter the body, they quickly bind with various plasma proteins, altering their surface properties (Panico et al. 2022). This change can obscure targeting ligands and hasten nanoparticle clearance, yet under certain conditions, it might boost nanoparticle uptake by tumour tissues (Deng et al. 2025).

The corona comprises a stable hard corona (tightly bound, long-residence proteins) and a dynamic soft corona (loosely bound, rapidly exchanging biomolecules), which together influence immune recognition and cellular uptake (Liam-Or et al. 2024). The formation of a protein corona critically governs nanomaterials clearance, as opsonization by complement proteins and immunoglobulins enhances recognition and uptake by the mononuclear phagocyte system (Tang et al. 2023). Recent studies further demonstrate that specific corona components, such as apolipoproteins and complement factors (*e.g.* C3), can directly modulate circulation half-life and cellular uptake pathways, highlighting that corona composition rather than total protein adsorption determines NP pharmacokinetics (Haripriyaa and Suthindhiran 2023; Hajipour et al. 2023; Mahmoudi et al. 2023; Han et al. 2026). Specifically, corona composition determines the binding of opsonins (*e.g.* immunoglobulins, complement proteins) versus dysopsonins (*e.g.* albumin), which directly affects recognition by phagocytic receptors (*e.g.* Fc receptors, scavenger receptors) on antigen-presenting cells (Abbina et al. 2020; Papini et al. 2020; Li et al. 2021). An opsonin-rich corona enhances Fc receptor-mediated phagocytosis and subsequent antigen processing, while a corona enriched with dysopsonins reduces uptake and prolongs circulation (Yang et al., 2026). This principle is well illustrated by gold nanorod studies: non-PEGylated, CTAB-capped nanorods are cleared from the bloodstream in less than 15 minutes, whereas PEGylation, which reduces the adsorbed protein corona, extends the circulation half-life to 19 h and promotes broader tissue distribution (Lin et al. 2022). Moreover, corona proteins can be processed and presented via MHC molecules, thereby modulating T cell activation, a critical link between nanoparticle design and adaptive immunity (Li et al. 2022; Tran and Roffler 2023; Shaw et al. 2025).

Notably, the composition of these protein coronas is highly dynamic and varies among patients, undermining the consistency of biological responses and treatment outcomes across different patient groups, and complicating efficacy predictions in clinical settings (Mahmoudi et al. 2023). Moreover, the primary molecular targets of many immunonanomedicines remain unidentified, and the intrinsic biological activity of nanomaterials further complicates the analysis and understanding of their therapeutic effects (Mahmoudi et al. 2023). However, accumulating evidence from recent studies reveals a previously underappreciated mechanism whereby nanomaterials can directly and selectively interact with drug target proteins via their intrinsic physicochemical properties, independent of conventional ligand modification (Shao et al. 2021; Zhang et al. 2022; Zhang et al. 2024; Liu et al. 2025; Liu et al. 2026). This target-specific nano-protein interaction paradigm introduces a mechanistic breakthrough for the rational design of nanotherapeutics with clearly defined molecular targets (Wu et al. 2024).

#### 2.2.2. Limitations in pharmacodynamic performance

Nanoscale immunotherapy offers precise delivery and control of immunomodulatory drugs via nanomaterials, enhancing anti-tumour immunity (Zhu and Li 2023). However, even with successful delivery, clinical studies show that only about 0.7% of systemically administered nanoparticles reach tumour sites, pharmacodynamic (PD) performance, *i.e.* the ability to elicit a durable and effective anti-tumour immune response, remains severely constrained by multiple tumour-intrinsic and host-related factors (Nguyen et al. 2024).

First, the tumour microenvironment (TME) imposes physical and immunosuppressive barriers that directly undermine PD outcomes. High interstitial pressure, a dense extracellular matrix (ECM), and rapid clearance in the TME hinder delivery efficiency (Xu et al. 2022). Beyond these physical barriers, specific TME components further impede therapeutic outcomes: (1) stromal density, the dense ECM and fibrotic stroma physically obstruct nanoparticle penetration and immune cell infiltration; (2) immune exclusion, T cells are often sequestered in peritumoral stromal regions, preventing their entry into tumour parenchyma; (3) hypoxia-driven immunosuppression, low oxygen tension upregulates HIF-1α, leading to PD-L1 expression, recruitment of regulatory T cells (Tregs), and secretion of immunosuppressive cytokines (*e.g.* IL-10, TGF-*β*) (Zhang et al. 2025; Huang et al. 2025). These multifaceted barriers and corresponding engineering strategies are systematically summarised in Table 1.

**Table 1.** Barrier–strategy mapping of nanoscale immunotherapy: A PK/PD-Informed framework.

Barrier type	Specific challenge (Derived from Text)	Underlying mechanism	Engineering strategy	Implementation considerations	Representative examples
Delivery Barrier Sindhwani et al. (2020); Mitchell et al. (2021); Zhang et al. (2024); Shi et al. (2025)	Extremely low tumour accumulation (~0.7–1%)	Rapid systemic clearance; limited extravasation	Nanocarrier optimisation (size, surface, targeting ligands)	Balance circulation time vs. tissue penetration	PEGylated nanoparticles, active targeting systems
Tumour Microenvironment Barrie**r** Banerjee et al. (2024); Chen et al. (2024); Luo et al. (2024); Zhong et al. (2026)	High interstitial pressure; dense ECM	Physical obstruction to penetration and diffusion	Microenvironment modulation (ECM degradation, pressure normalisation)	Combine with penetration enhancers	ECM-degrading enzymes, stromal modulation
Biological Clearance Barrier Luo et al. (2022); Anchordoquy et al. (2024); Zhang et al. (2025)	Uptake by mononuclear phagocyte system (MPS)	Preferential accumulation in liver and spleen	Stealth design (surface engineering, protein corona control)	Minimise opsonization and macrophage recognition	Surface-functionalized nanoparticles
Size-Dependent PK Barrie**r** Xu et al. (2023); Kim et al. (2024)	Size-dependent biodistribution and clearance	Larger particles retained in liver; ultrasmall cleared renally	Size optimisation and dynamic size transformation	Tailor size for specific PK profiles	Ultrasmall nanoparticles, transformable nanocarriers
Immune Activation Barrie**r** Goldberg (2019); Wang et al. (2024); Butterfield and Najjar (2024)	Overactivation of innate immunity; insufficient adaptive response	Imbalanced immune modulation	Immune programming (co-delivery, sequential activation)	Coordinate innate and adaptive immunity	Combination immunotherapy platforms
T Cell Dysfunction Wu et al. (2022); Admasu and Yu (2025)	T cell exhaustion	Chronic antigen exposure, immunosuppressive signals	Checkpoint modulation, T cell rejuvenation	Combine with checkpoint inhibitors	PD-1/PD-L1 blockade systems
Tumour Heterogeneity Noor et al. (2024); Wu et al. (2026)	Inter-patient variability; lack of predictive biomarkers	Genetic and immune heterogeneity	Personalised nanomedicine; biomarker-guided design	Stratify patients based on response profiles	Precision nanotherapy
Pharmacokinetic Variability Ferdosi et al. (2022); Tan et al. (2024)	Large inter-individual variability in half-life and distribution	Protein corona formation; nano–bio interactions	PK-informed design and modelling	Integrate physicochemical properties with PK prediction	Data-driven nanomedicine

Second, PD performance is limited by suboptimal activation kinetics and unsustainable adaptive immunity. Some therapies overly activate innate immunity without sustaining adaptive immunity, and T cell exhaustion remains unresolved (Yu et al. 2026). The immunosuppressive TME further raises the threshold for immune activation, requiring stronger or more prolonged stimulation to achieve a therapeutic effect (Ang et al. 2026). However, excessive activation risks off-target toxicity and cytokine storms, creating a narrow therapeutic window.

Third, patient-specific tumour and immune heterogeneity, coupled with a lack of reliable predictive biomarkers, further complicate treatment efficacy (Passaro et al. 2024). The dose-effect relationship in nano-immunotherapy is often nonlinear, as it integrates drug release kinetics, immune activation threshold, and dynamic feedback from the TME. Controllable stimulus-responsive systems can enable spatiotemporally regulated release, but insufficient or premature release reduces efficacy, whereas delayed or excessive release heightens toxicity. Thus, optimising PD outcomes requires coordinated regulation of dosage, release behaviour, and immune activation.

Concerns about the long-term biosafety and stability of nanomaterials, along with the complexity of large-scale preparation and clinical trial design, also impede clinical translation (Zhang et al. 2023). Nonetheless, advancements in nanomaterial design, personalised treatment strategies, and more relevant preclinical models continue to support the potential of nanoscale immunotherapy for clinical applications (Jiang et al. 2021).

#### 2.2.3. Long-term safety

The long-term safety of nanoimmunotherapy remains a major challenge due to slow clearance and off-target organ accumulation (Wang et al. 2025). These challenges are not unique to nanocarrier systems but also apply to nanomaterial-based immune adjuvants, nano-combined therapeutic platforms, and nano-enabled cell therapies. The nanoscale physicochemical properties of these systems strongly shape their *in vivo* biodistribution and clearance routes (Cabral et al. 2024).

The metabolic fate of nanomedicines is largely determined by material composition. In many cases, inorganic nanomaterials exhibit slow degradation and prolonged retention *in vivo*, whereas organic nanomaterials are generally more biodegradable and can be cleared more readily (Wang et al. 2021). Regarding organ accumulation, nanoparticles typically localise to the liver and spleen: particles larger than 200 nm are more readily sequestered by hepatic Kupffer cells, while efficient renal elimination is generally restricted to ultrasmall nanoparticles with a hydrodynamic diameter of smaller than 6 nm (Xu et al. 2023). Persistent nanomaterial accumulation may promote chronic inflammation, perturb immune homoeostasis, and contribute to organ toxicity (Xuan et al. 2023). Beyond these general biosafety concerns, immunotoxicity represents an additional critical challenge in nanoimmunotherapy (Hofer et al. 2022). Off-target immune activation can result from nonspecific uptake by immune cells or unintended interactions with circulating biomolecules, potentially triggering complement activation and inflammatory responses (Lee et al. 2023). Moreover, excessive immune stimulation may lead to cytokine-related toxicity, characterised by uncontrolled release of pro-inflammatory cytokines and systemic adverse effects, particularly in the context of immunostimulatory nanocarriers (Kang et al. 2025). Long-term accumulation in organs such as the liver and spleen may further exacerbate chronic inflammation and immune imbalance (Hofer et al. 2022). Therefore, improving metabolism and clearance, while incorporating safety-by-design strategies, such as the use of biodegradable materials, controlled immune activation, and comprehensive long-term toxicity evaluation, is essential for maximising therapeutic efficacy and minimising biosafety risks (Duan et al. 2025). Overall, addressing long-term biosafety concerns remains critical for the successful clinical translation of diverse nanoimmunotherapy platforms (Zhang et al. 2022).

## 3. Strategies for clinical translation

### 3.1. Mechanism-driven and target-defined design

To realise the full potential of nanoimmunotherapy, a mechanism-guided ‘barrier-strategy’ framework is essential. This framework directly links biological challenges to engineering solutions. Specifically, addressing PK variability requires surface engineering and corona modulation; overcoming PD limitations demands improved tumour penetration, controlled release, and balanced immune activation; and mitigating safety concerns calls for biodegradable and clearance-optimised designs (De et al. 2025). Key parameters, including size, charge, ligand functionalization, and stimulus-responsiveness, must be co-optimised (Fan et al. 2023). Targeting strategies include both active targeting (via ligands such as antibodies and peptides) and passive targeting (by tuning physicochemical properties like size and shape), with AI and organoid systems helping to align carrier functions with disease mechanisms such as inflammation and oxidative stress (Srinivasarao and Low 2017; Chen et al. 2023; Han et al. 2024; Shi et al. 2024). Stimulus-responsive designs enable site-specific activation based on microenvironmental signals, including acidic pH and elevated reactive oxygen species (Wei et al. 2022; Chen et al. 2023). To enhance biocompatibility, degradable materials such as PLGA and PEGylation are employed to reduce toxicity (Su and Kang 2020).

In this context, AI facilitates data-driven design by predicting nano-bio interactions and optimising formulations. Recent advances demonstrate that AI-assisted frameworks can move beyond conceptual optimisation toward quantitative nanoparticle design (Han et al. 2025). Machine learning and deep learning models are increasingly used to predict key parameters such as particle size, drug loading, and biodistribution by integrating physicochemical and biological datasets, enabling rational rather than trial-and-error design (Sheikh and Jirvankar 2024). Notably, integrated platforms combining microfluidics, high-content screening, and active learning have been shown to iteratively optimise nanoparticle formulations, achieving up to a ~15 fold enhancement in cellular uptake within only two design cycles (Ortiz-Perez et al. 2024). In parallel, AI models can predict nanoparticle pharmacokinetics and clearance, providing mechanistic insights into in vivo behaviour (Khakpour et al. 2025). Despite these advances, challenges such as limited high-quality datasets and model interpretability remain barriers to clinical translation. Multi-omics analysis can monitor real-time biocompatibility changes and predict metabolic pathways and risks (Yang et al. 2026), while-AI driven modelling further supports the prediction of nano-bio interactions. Ultimately, interdisciplinary collaboration across materials science, immunology, and computer science, combined with integrated diagnostics and therapeutics, enhances our understanding of nanomedicine mechanisms and reduces clinical translation risks (Das et al. 2025). Strategies such as co-delivery systems and microenvironment responsive nanocarriers illustrate how these principles can be effectively translated into therapeutic platforms (Fan et al. 2023; Su and Qiu, 2025).

### 3.2. Rational nanoparticle-enabled pharmacodynamics engineering

Nanomedicines face challenges like rapid systemic clearance, poor tumour penetration, and drug resistance in single therapies (Xu et al. 2023). To overcome these, multi-dimensional synergy strategies, including AI and interdisciplinary integration, enhance therapeutic efficacy (Habeeb et al. 2025). Nanomedicines combine chemotherapy, phototherapy (PDT/PTT), and immunotherapy, with nanocarriers enabling simultaneous drug delivery. For example, a drug may also function as a photothermal therapy agent, facilitating controlled release and generating reactive oxygen species, which synergistically reduce drug resistance (Su et al. 2021). ATR (active transport and retention) technology improves tumour-targeting by regulating lymphatic transport and enhancing drug retention (Wu et al. 2025). Multi-omics analysis aids in optimising these technologies by decoding lymphatic vessel regulation. In immunotherapy, nanocarriers deliver immunomodulators and siRNA to remodel the immune microenvironment, converting M2 macrophages to M1 and enhancing therapeutic effects (Guo et al. 2025). Theranostic strategies, integrating imaging capabilities like fluorescence and MRI, enable real-time treatment monitoring, supporting personalised medicine (Liu et al. 2025). Multi-strategy synergy and advanced technologies maximise the efficacy of nanomedicines, overcoming delivery challenges and optimising treatment regimens (Huayamares et al. 2024). This adaptability, combined with advanced technologies' predictive abilities, effectively addresses delivery challenges. Ultimately, through multi-strategy synergy, interdisciplinary integration, and advanced technology empowerment, nanomedicine efficacy is maximised (Zhang et al. 2019; Jannatun et al. 2023; Liu et al. 2024).

### 3.3. Designing safe metabolic optimisation strategies

To mitigate organ toxicity from prolonged nanomaterial retention, biodegradable materials like chitosan and polylactic acid are preferred, as their breakdown products are naturally excreted, minimising accumulation risk. Non-degradable materials, such as black phosphorus (BP), require modifications like quantum dots or ultra-thin nanosheets to enhance lysosomal hydrolysis (Shao et al. 2021). For example, copper indium phosphorus sulphur nanosheets (CIPS) are cleared from the lungs within a week, reducing liver and spleen accumulation. AI predicts degradation kinetics, while organoids simulate human metabolism for optimisation.

Bionic strategies like PEG modification and cell membrane coating enhance biocompatibility, minimising immune inflammation and non-specific organ accumulation. PEG-modified gold (Au) nanomaterials decrease protein adsorption and reduce cytotoxicity (Wang et al. 2025). Multi-omics technologies, combined with AI, optimise size and shape for better *in vivo* distribution.

Environment-responsive materials release drugs exclusively in the acidic tumour microenvironment, minimising off-target effects. Controlled-release technology ensures metabolic safety and precise drug delivery. Toxicological research and long-term metabolic tracking are essential for safe translation, while developing standardised metabolic evaluation systems improves safety predictions (Tagaras et al. 2024). Incorporating metabolic concepts and quality control in production processes balances therapeutic efficacy with safety. Developing a standardised metabolic evaluation system to simulate human metabolism over time can improve the accuracy of safety predictions.

## 4. Clinical progress, market trends, and future prospects

Despite over 60 nanomedicines having been clinically approved worldwide, including LNP-based RNA therapeutics (*e.g.* Onpattro®, 2018; Comirnaty®, 2020) and established formulations such as Doxil® (1995) and Abraxane® (2005) (Croitoru et al. 2024), most remain first- or second-generation systems with limited active targeting capabilities (Herdiana 2025). Several next-generation candidates (e.g. BIND-014, terminated ~2016; CRLX101, halted ~2021) failed in late-stage trials due to insufficient efficacy and lack of significant survival benefit, highlighting persistent translational challenges such as biological heterogeneity, suboptimal delivery efficiency, and safety concerns (Al Shaer et al. 2026). In parallel, recent advances in immunotherapy and delivery technologies are providing important translational references for nanoimmunotherapy. For example, Opdivo Qvantig improves the clinical delivery format of immune checkpoint blockade, highlighting the value of formulation and administration-route innovation for immunotherapy optimisation (Timmins 2025). mRNA vaccines supported by lipid nanoparticles, such as EVM16, have shown encouraging immunogenicity. (Agrawal et al. 2025). Additionally, the approval of AMTAGVI™ and the exploration of ‘armored CAR T’ therapies demonstrate the progress of cell-based immunotherapy for solid tumours, while nanoengineering strategies may further contribute to cell modification, in vivo delivery, and tumour-microenvironment modulation in this field (Parums 2024). Cytokine-based approaches, such as Anktiva and its potential integration with nano-delivery systems to improve cytokine localisation and reduce systemic toxicity, are also attracting attention (Zheng et al. 2022).

More recent advancements between 2023 and 2026 are further reshaping the nano-immunotherapy landscape. Bionic nanoplatforms, including membrane-coated nanoparticles and exosome-mimicking vesicles, enhance immune compatibility, circulation stability, and targeting (Xu et al. 2026). Recent studies have further demonstrated that tumour microenvironment–responsive and biomimetic nanoplatforms can integrate hypoxia modulation, imaging guidance, and synergistic therapeutic modalities, thereby improving therapeutic precision and efficacy (Wang et al. 2024; 2025). In addition, exosome-inspired and biomimetic delivery systems have been extended to immune-related diseases beyond cancer, highlighting their broader potential for immune regulation (Wang et al. 2024; Yang et al. 2025). Meanwhile, mRNA/LNP systems have matured into clinically relevant platforms for antigen delivery and combination immunotherapy, with LNPs poised to lead market competition by reducing research, development, and regulatory risks (Zhu et al. 2025). Moreover, emerging strategies such as carrier-free nanoparticles, bio-conjugated nanostructures, and physical modulation approaches are expanding the design space for precise delivery, tumour imaging, and immune microenvironment remodelling (Zhang et al. 2025; Liang et al. 2026; Dong et al. 2026). Nevertheless, most nano-immunotherapy candidates remain in early-stage development, and the gap between clinical promise and real-world implementation persists.

Future growth is expected in personalised vaccines, combination therapies, and solid tumour cell therapy. Advances in AI-driven, mechanism-focused approaches will drive breakthroughs, with technologies like Cross-linking Mass Spectrometry (CL-MS) and Quartz Crystal Microbalance with Dissipation Monitoring (QCM-D) clarifying ‘material-target’ interactions (Liu and Zeng 2025). AI technology is increasingly used to optimise nanoparticle design and predict nano-bio interactions (*e.g.* protein corona, biodistribution), steering the field toward biomimetic and data-driven strategies. Delivery engineering will focus on overcoming EPR effect limitations and improving spatial distribution and immune targeting. At the industrial level, standardised production and quality control systems are crucial. Advanced translational tools such as humanised tumour models and organ-on-a-chip can reduce preclinical and clinical uncertainties. Interdisciplinary collaboration will help transition technologies from lab to routine clinical practice, narrowing the translational gap.

## 5. Conclusion

Nanoimmunotherapy holds considerable promise for cancer treatment; however, its clinical translation continues to be limited by challenges related to PK and PD, and long-term safety. Addressing these issues calls for a shift toward mechanism-driven design, with a focus on nano-bio interactions and tumour microenvironment regulation. Optimising PK/PD performance is essential and can be achieved through improved delivery systems, controllable drug release, and balanced immune activation. Moreover, safety considerations should be integrated via biodegradable material design and long-term toxicity assessments. Emerging technologies such as AI and multi-omics are advancing predictive therapeutic strategies. In parallel, sophisticated translational models are needed to bridge the gap between preclinical research and clinical practice. Ultimately, integrating mechanistic insights with engineering development and clinical validation will be key to advancing nanoimmunotherapy toward real-world application. Overall, the integration of mechanistic insights with engineering innovation and rigorous clinical validation will be essential to advance nanoimmunotherapy toward effective real-world implementation.

## Data Availability

Data sharing is not applicable to this article as no new data were created or analysed in this study.
